# A multi-source information fusion approach in tunnel collapse risk analysis based on improved Dempster–Shafer evidence theory

**DOI:** 10.1038/s41598-022-07171-x

**Published:** 2022-03-07

**Authors:** Bo Wu, Weixing Qiu, Wei Huang, Guowang Meng, Jingsong Huang, Shixiang Xu

**Affiliations:** 1 College of Civil Engineering and Architecture, Guangxi University, 100 University Road, Nanning, 530004 Guangxi China; 2 School of Civil and Architectural Engineering, East China University of Technology, Nanchang, 330013 Jiangxi China; 3 School of Architectural Engineering, Guangzhou City Construction College, Guangzhou, 510925 Guangdong China

**Keywords:** Natural hazards, Civil engineering, Information technology

## Abstract

The tunneling collapse is the main engineering hazard in the construction of the drilling-and-blasting method. The accurate assessment of the tunneling collapse risk has become a key issue in tunnel construction. As for assessing the tunneling collapse risk and providing basic risk controlling strategies, this research proposes a novel multi-source information fusion approach that combines Bayesian network (BN), cloud model (CM), support vector machine (SVM), Dempster–Shafer (D–S) evidence theory, and Monte Carlo (MC) simulation technique. Those methods (CM, BN, SVM) are used to analyze multi-source information (i.e. statistical data, physical sensors, and expert judgment provided by humans) respectively and construct basic probability assignments (BPAs) of input factors under different risk states. Then, these BPAs will be merged at the decision level to achieve an overall risk evaluation, using an improved D–S evidence theory. The MC technology is proposed to simulate the uncertainty and randomness of data. The novel approach has been successfully applied in the case of the Jinzhupa tunnel of the Pu-Yan Highway (Fujian, China). The results indicate that the developed new multi-source information fusion method is feasible for (a) Fusing multi-source information effectively from different models with a high-risk assessment accuracy of 98.1%; (b) Performing strong robustness to bias, which can achieve acceptable risk assessment accuracy even under a 20% bias; and (c) Exhibiting a more outstanding risk assessment performance (97.9% accuracy) than the single-information model (78.8% accuracy) under a high bias (20%). Since the proposed reliable risk analysis method can efficiently integrate multi-source information with conflicts, uncertainties, and bias, it provides an in-depth analysis of the tunnel collapse and the most critical risk factors, and then appropriate remedial measures can be taken at an early stage.

## Introduction

The highways are extremely important infrastructures for most countries. It ensures communication and development between different regions, especially in the mountains and hilly areas. Most of the surrounding rocks of highway tunnels are mainly hard rock mass, and the geological conditions of the crossing sections are complex and changeable^1^. Hard rock tunnels are mostly constructed by drilling and blasting. Due to various risk factors in the complex project environment, safety violations often occur in highway tunnel construction. The collapse is one of the most frequent and harmful geological hazards during the construction of a tunnel. Because the collapse was sudden and instantaneous, it was difficult to predict and the construction workers did not have enough time to escape. Once the tunnel collapse occurs, it may cause serious economic losses, construction delays, and even human casualties. Therefore, it is necessary to research the risk mechanism of tunnel collapse by considering the accident scenario and safety analysis, aiming to provide decision support for assuring the safety of tunnel construction.

In recent years, a lot of research work has been carried out in tunnel collapse risk assessment. Zhou^2^ proposed a method for tunnel collapse risk analysis based on the fuzzy Analytic Hierarchy Process. He discussed the collapse mechanism of mountain tunnels and proposed a list of risk factors for tunnel collapse. The Bayesian network is used to conduct a quantitative analysis of safety risks in the Wuhan Yangtze River Metro Tunnel^3^. Wu et al.^4^ proposed an evaluation method based on a dynamic Bayesian network to provide a real-time dynamic risk assessment for tunnel construction. An optimization method for the preliminary support parameters was proposed based on the genetic algorithm (GA) and combined covariance Gaussian process regression (CCGPR) coupled algorithm presented to provide a complete information-based construction method for tunnel engineering^5^. There are also many studies using artificial intelligence for risk assessment to realize the automation and intelligence of assessment. Pan^6^ used artificial intelligence to monitor the entire life cycle of real complex projects. The artificial neural networks are used to assess the risk of shield drilling under severe ground conditions such as squeezing grounds^7^.

However, since the above evaluation methods only focus on a single information source, the reliability and accuracy of the security risk assessment cannot be guaranteed. Incomplete consideration of information can lead to inaccurate assessment results, which can not provide accurate recommendations to decision makers^8^. This would defeat the purpose of the risk assessment. In comparison, the fusion model can greatly improve the accuracy of prediction results due to it has a better understanding of risk factors^9^. For example, a fusion model is proposed to predict the risk of water inrush disasters^10^. The fusion of sensor data and simulation data improves the accuracy of the structural safety risk assessment^9^. Nowadays, there has been an increasing interest in the development of modern information technology and Internet technology, which makes the processing and analysis of data from multiple sources particularly important. The data fusion technology may prove to be more helpful to meet the security risk management needs of the tunnel construction than point-based methods^11^. Over the years, various information fusion studies have been proposed, such as Dempster–Shafer (D–S) evidence theory^12,13^, maximum entropy method^14^, rough sets^15^, etc. Among the above information fusion methods, D–S evidence theory is an effective and commonly used method in the field of information fusion. However, the traditional D–S theory of evidence has two disadvantage that may not be appropriate in practical situations. (1) When multiple sources of information are evaluated differently, D–S theory gives fusion results that are contrary to common sense. (2) The probability distribution is based on a user-defined function or distribution, which is too ideal for practical purposes. To solve the above problems, This research proposes a novel risk assessment approach that integrates Monte–Carlo (MC) simulation technique, normal cloud model (CM), Bayesian networks (BN), probabilistic support vector machine (SVM), and improved D–S evidence theory. The tunneling collapse risk probability distribution is obtained by analyzing different information sources with different models. Finally, the judgment of each model is fused to give the overall collapse risk result. This model aims to achieve the following goals: (1) Constructing models to estimate the collapse risk according to the expert judgment, monitoring data, and tunneling collapse database; (2) The judgment of the models is fused to get the final collapse risk assessment result; (3) Evaluating the performance of the models to quantify the quality of judgment.

## Literature review

### Dempster–Shafer (D–S) evidence theory

Information sources are usually divided into three categories, namely statistical data, physical sensors, and expert judgment provided by humans^14^. Among them, statistical data and physical sensors are called hard information. Humans act as soft sensors and execute decision-making processes through a web-based system^16^. Regarding evidence, each source of information constitutes all the evidence on which the decision is based^17^. In the complex decision-making process, how to compose multiple sources of evidence that may conflict with each other has become a challenging task. So far, over the years, various information fusion researches have been proposed, such as rough set^15,18^, Dempster–Shafer (D–S) evidence theory^8,17^, maximum entropy approach^14^, and others. Among the above-mentioned information fusion methods, D–S evidence theory is an effective and common method in the field of information fusion. Pan et al.^9^ proposed a risk analysis method based on SVM and D–S evidence theory to fuse different monitoring data, in order to evaluate the structural health status. Zhang et al.^19^ developed a novel safety risk assessment method based on D–S evidence theory and the cloud model to perceive the safety risk of buildings adjacent to the tunneling excavation.

However, the traditional D–S evidence theory cannot deal with highly conflicting evidence and will lead to unexpected and counter-intuitive results and make the evidence fusion approach insignificant^19^. In order to minimize the negative effect of high-conflict evidence, this paper adopted an improved D–S evidence theory by combining the weighted mean rule and the D–S evidence theory to solve the above problem.

### Classification method

For classification problems, support vector machines (SVM) and artificial neural networks (ANN) are the two main supervised learning algorithms in the field of machine learning^20^. Although ANN has provided a powerful tool for the research on tunnel construction^21,22^. There are still limitations such as long calculation time, spatial disasters, local minima, overfitting, etc.^6,23^. Due to the small sample of training data for the tunneling collapse case in this paper, classification using neural networks will be prone to overfitting. The support vector machines (SVM), as a method parallel to artificial neural networks (ANN), is a machine learning method established based on the principle of structural risk minimization and the statistical learning theory for a small sample. The SVM has higher accuracy in a small number of training data predictions. Therefore, this article attempts to use SVM to process statistical data for collapse risk assessment.

## Methodology

**Figure 1:**
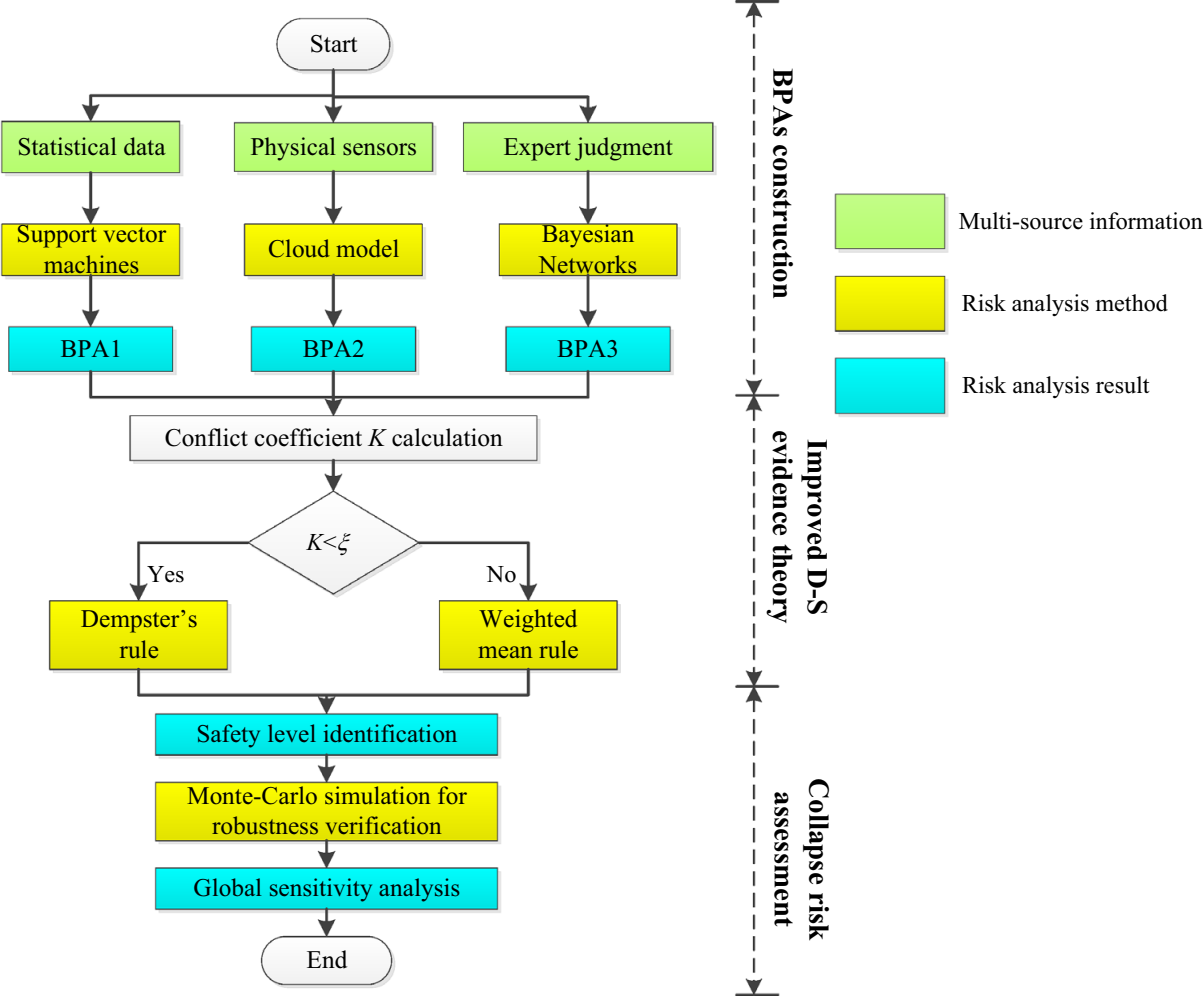
Flowchart of the proposed hybrid method for multi-source information fusion decision.

In order to improve the credibility and robustness of the tunnel collapse risk evaluation, a new hybrid multi-source information fusion method is proposed. Figure 1 is a flowchart of the tunnel collapse risk analysis method in this paper. In the developed method, all available data from the construction process is collected for risk analysis to improve the accuracy and robustness of the assessment results. In the process of data fusion, an improved D–S evidence theory is utilized to refine and synthesize different classification results generated from probabilistic models. According to the characteristics of different information sources, choose the corresponding probability model.

For statistical data, since the statistical data has been classified, SVM with the advantage of small sample classification is used for risk assessment. For physical sensors, quantitative monitoring data needs to be mapped to qualitative collapse risk values. The CM combines fuzzy mathematics and probability theory to map qualitative concepts and quantitative data and is therefore used to process monitoring data during the constructs of BPAs. For expert judgment provided by humans, Bayesian Networks (BN) is used to investigate causal relationships between tunnel collapse and its influential variables based upon the risk/hazard mechanism analysis and expert scores.

In collapse risk assessment, the MC simulation can conduct risk analysis by constructing a calculation model containing a series of inherent uncertain variables^24^. It can estimate all possible decision results and evaluate the impact of risks in an uncertain environment^25^. The MC simulation is adopted to simulate measurement and human error, proving the robustness of the hybrid approach. A typical hazard concerning the tunnel collapse in the construction of the Fujian Jinzhupa Tunnel in China is presented as a case study. The results demonstrate the feasibility of the proposed approach and its application potential.

### BPA construction

#### Normal cloud model

The normal cloud model is a new cognition model of uncertainty, proposed by Li et al.^26^. It can synthetically describe the randomness and fuzziness of elements and implement the uncertain transformation between a qualitative concept and its quantitative value. The normal CM can be determined by numerical characteristics (*Ex*, *En*, *He*). The Expectation “*Ex*” is the expectation of the cloud droplets in the universe of discourse and the typical sample of a qualitative concept. The Entropy “*En*” is the entropy of “*Ex*”, representing the uncertainty measurement of a qualitative concept. Hyper-entropy “*He*” represents the uncertainty degree of Entropy “*En*”*.*

Let *X* be the universe of discourse and *B* be a qualitative concept connected with *X*. If there is a number *x*, (1)$x \in X$, (2) *x* is a random instantiation of concept *B*, (3) *x* satisfies Eq. (1), the grade of a certain degree of *x* belonging to concept *B* satisfies Eq. (2)^26^:

1 $$ \left\{ {\begin{array}{*{20}l} {x \sim N\left( {Ex,En^{{\prime}{2}} } \right)} \\ {En^{\prime} \sim N\left( {En,He^{2} } \right)} \\ \end{array} } \right. $$

2 $$ \mu \left( x \right) = e^{{ - \frac{{\left( {x - Ex} \right)^{2} }}{{2\left( {En^{\prime}} \right)^{2} }}}} $$

The tunneling collapse risk assessment is a multi-source information decision-making problem under uncertain conditions. Various tunnel collapse risk factors *B*_*i*_ are analyzed in the decision-making process. In order to explore useful information from multiple sources, each risk factor should be further divided into different risk states *B*_*ij*_ (*i* = 1, 2, …, *M*; *j* = 1,2, …, *N*). Each risk state can correspond to a specific double limit interval, denoted as [*b*_*ij*_(*L*), *b*_*ij*_(*R*)]. The conversion from the double limit interval [*b*_*ij*_(*L*), *b*_*ij*_(*R*)] to the normal cloud model $\left( {Ex_{ij} ,En_{ij} ,He_{ij} } \right)$ can be achieved by Eq. (3)^26^.

3 $$ \left\{ {\begin{array}{*{20}l} {Ex_{ij} = \frac{{b_{ij} (L) + b_{ij} (R)}}{2}} \\ {En_{ij} = \frac{{b_{ij} (R) - b_{ij} (L)}}{6}} \\ {He_{ij} = h} \\ \end{array} } \right.\begin{array}{*{20}l} , & {\left( {i = 1,2, \, \ldots ,M;j = 1,2, \, \ldots ,N} \right)} \\ \end{array} $$

where, “*Ex*_*ij*_” is the expectation; “*En*_*ij*_” is the entropy of “*Ex*_*ij*_”, “*He*_*ij*_” is the Hyper-entropy. The range of the constant “*h*” is from 0 to “*En*_*ij*_” which is adapted to reflects the uncertainty degree of those factors.

In the CM framework, the correlation can measure the relative membership between the observed value *b*_*ij*_ of the factor *B*_*i*_ and the cloud model of a specific risk state *B*_*ij*_. The measurement of BPAs under different risk states of influential factors can be obtained by Eq. (4)^19^.

4 $$ \left\{ {\begin{array}{*{20}l} {m_{i} \left( {B_{j} } \right) = \exp \left( { - \frac{{\left( {x_{i} - Ex_{ij} } \right)^{2} }}{{2\left( {En_{ij}^{\prime } } \right)^{2} }}} \right)} \\ {m_{i} \left( \Phi \right) = 1 - \sum\limits_{j = 1}^{N} {m_{i} \left( {A_{j} } \right)} } \\ \end{array} } \right.\begin{array}{*{20}l} , & {\left( {i = 1,2, \, \ldots ,M;j = 1,2, \, \ldots ,N} \right)} \\ \end{array} $$

where, *m*_*i*_(*B*_*j*_) is the belief measure; $En^{\prime}$ represents a random number that satisfies $En^{\prime} \sim N\left( {En,He^{2} } \right)$, and *m*_*i*_(Φ) represents the BPAs value in uncertain situations, that is, the focus element cannot be determined under the indicator *B*_*i*_, so all elements are included.

#### Probabilistic SVM

The traditional linear SVM performs linear division by a hyperplane. This hyperplane is found by maximizing the separation margin, which is the distance between the hyperplane and the closest data point. The kernel function is used to map the original data from a low-dimensional space to a feature space with a high-dimensional space, which can obtain better classification accuracy. Besides, the penalty parameter *C* of the error term also plays a key role in classification accuracy. A high value of *C* means a strict classifier that does not admit many misclassified points^27^. The discrimination function is:

5 $$ f\left( x \right) = sign\left[ {\left( {\sum\limits_{i = 1}^{m} {\alpha_{i} y_{i} K\left( {x_{i} ,x} \right)} } \right) + b} \right] $$

where *m* is the size of the training data set, *α*_*i*_ represents Lagrange multipliers, *K*(*x*_*i*_, *x*) is a kernel function, and *b* is a threshold parameter based on the training set.

The linear SVM only gives one class prediction output that will be either yes or no. To extract the associated probabilities from SVM outputs, several methods have been proposed. This research chooses Platt's approach, which uses the Sigmoid function to map the output of the SVM to the interval [0, 1], as given by Eq. (6)^9^.

6 $$ P\left( {y = \left. 1 \right|x} \right) \approx P_{ab} \left( {f\left( x \right)} \right) = \frac{1}{{1 + e^{{\left( {af\left( x \right) + b} \right)}} }} $$

where *a* and *b* are the parameters computed from the minimization of the negative log-likelihood function on a set of training examples:

7 $$ \begin{gathered} \mathop {\min }\limits_{z = (A,B)} F(z) = - \sum\limits_{i = 1}^{l} {\left( {t_{i} \log (p_{i} ) + (1 - t_{i} )\log (1 - p_{i} )} \right)} , \hfill \\ \left\{ {\begin{array}{*{20}l} {\begin{array}{*{20}l} {t_{ + } = \frac{{N_{ + } + 1}}{{N_{ + } + 2}}} & {} \\ \end{array} } \\ {\begin{array}{*{20}l} {t_{ - } = \frac{1}{{N_{ - } + 2}}} & {} \\ \end{array} } \\ \end{array} } \right.i = 1,2, \ldots ,l \hfill \\ \end{gathered} $$

where *t*_*i*_ is the new label of the classes: + 1 becomes *t*_+_ and − 1 becomes *t*_−_, *N*_+_ and *N*_−_ are the number of points that belong to class 1 and class 2 respectively.

#### Bayesian network

The Bayesian network (BN) is a combination of two different mathematical areas, the probability theory, and graph theory. It consists of several conditional probability tables (CPT) and a directed acyclic graph (DAG)^28^. A BN model with n nodes can be represented as $B\left\langle {G,\Theta } \right\rangle$, where G stands for a DAG with n nodes and Θ is defined as the CPT of the BN model. A general BN intuitively represents a complex network with *n* nodes and direct edges. The nodes $\left\{ {X_{1} , \cdots ,X_{n} } \right\}$ in the graph are labeled by related random variables. The directed edges between nodes represent the relationship between variables. Each node is attached to a CPT that contains the conditional probability of the parent node.

Assuming $parents\left( {X_{i} } \right)$ is the parent nodes of $X_{i}$ in DAG, the conditional probability distribution of $X_{i}$ is defined as $P\left( {\left. {X_{i} } \right|parents(X_{i} )} \right)$. The calculation of *P*(*x*) can be written as Eq. (8)

8 $$ P\left( x \right) = P\left( {X_{1} , \cdots ,X_{n} } \right){ = }\prod\limits_{{X_{i} \in \left\{ {X_{1} , \cdots ,X_{n} } \right\}}} {P\left( {\left. {X_{i} } \right|parents(X_{i} )} \right)} $$

### Improved D–S evidence theory

In this paper, the D–S theory is used to combine multi-source information to obtain the tunnel collapse risk. Dempster’s combinational rule for multiple evidence is calculated with Eq. (9)^19^.

9 $$ \left\{ {\begin{array}{*{20}l} {m\left( B \right) = \left\{ {\begin{array}{*{20}c} {\begin{array}{*{20}l} {\frac{1}{1 - K}\sum\limits_{{B_{i} \cap B_{i} \cap \ldots \cap B_{k} = B}} {m_{1} \left( {B_{i} } \right)m_{2} \left( {B_{j} } \right) \ldots m_{l} \left( {B_{k} } \right),} } & {\forall B \subseteq \Theta ,B \ne \emptyset } \\ \end{array} } \\ {\begin{array}{*{20}l} {0,} & {B = \emptyset } \\ \end{array} } \\ \end{array} } \right.} \\ {K = \sum\limits_{{B_{i} \cap B_{i} \cap \ldots \cap B_{k} = \emptyset }} {m_{1} \left( {B_{i} } \right)m_{2} \left( {B_{j} } \right) \ldots m_{l} \left( {B_{k} } \right) < 1} } \\ \end{array} } \right. $$

where *K* is defined to be the normalization factor. *l* is the number of evidence pieces in the process of combination, and *i*, *j*, *k* denotes the *i*th, *j*th, and *k*th hypothesis, respectively.

When the value of *K* is close to 1, there will be a high conflict, which means that Dempster's evidence aggregation rule will be meaningless. To deal with high-conflict evidence, this paper proposed a hybrid combination rule by combining the weighted mean rule and the Dempster’s rule. This article will use a threshold *ξ* to indicate high evidence conflicts. When *K* is greater than *ξ*, there is high evidence conflict, and the D–S evidence theory will be replaced by the weighted mean rule, as shown in Eq. (10)^19^. In this research, the value of the threshold *ξ* is defined to be 0.95^19^.

10 $$ \left\{ {\begin{array}{*{20}l} {d = \sum\limits_{j = 1}^{j = l} {\sqrt {\sum\limits_{k = 1}^{k = L} {\left( {m_{i} \left( {B_{k} } \right) - m_{j} \left( {B_{k} } \right)} \right)^{2} } } } } \hfill \\ {w_{i} = \frac{{d_{i}^{ - 1} }}{{\sum\limits_{i = 1}^{i = l} {d_{i}^{ - 1} } }}} \hfill \\ {\left\{ {\begin{array}{*{20}c} {m_{i}^{*} \left( {B_{k} } \right) = w_{i} \cdot m_{i} \left( {B_{k} } \right)} \\ {m_{i} \left( \Theta \right) = 1 - \sum\limits_{k = 1}^{L} {m_{i}^{*} \left( {B_{k} } \right)} } \\ \end{array} } \right.} \hfill \\ \end{array} } \right. $$

where *l* and *L* are the numbers of evidence and the number of hypotheses, respectively, and *k* is the *k*th hypothesis.

### Tunnel collapse risk assessment

The collapse risk assessment can provide support for construction decision-making on site. Once the collapse risk drops to a high-risk level, certain precautions can be taken before the tunnel collapses. After multiple information sources are fused at the decision-making level, the result of tunnel collapse risk assessment depends on the maximum value of BPAs, as shown in Eq. (11). The confidence indicator $m_{i} \left( \Theta \right)$ is designed to measure the credibility of the fusion result.

11 $$ \left\{ {\begin{array}{*{20}c} {m\left( {B_{w} } \right) = \max \left\{ {m\left( {B_{i} } \right)} \right\}} \\ {\begin{array}{*{20}c} {m\left( \Theta \right) < \theta } & {\theta { = }0.1} \\ \end{array} } \\ \end{array} } \right. $$

where *B*_*i*_ denotes collapse risk levels, *B*_*w*_ indicates the probability of different risk levels $m\left( B \right) = \left\{ {m\left( {B_{1} } \right),m\left( {B_{2} } \right),...,m\left( {B_{n} } \right),m\left( \Theta \right)} \right\}$.

The sensitivity analysis of the tunneling collapse risk factors is proposed to reveal the sensitivity of system performance to small changes in risk factors. Up to now, some sensitivity analysis methods have been proposed^29^. To consider the nonlinearity and interaction relationship between risk factors, this paper adopts global sensitivity analysis (GSA). Spearman's rank correlation coefficient (a GSA measure) does not depend on distributions with a similar shape or being linearly related. The *GSA* measurement of the *i*th input factor *C*_*i*_ can be calculated by Eq. (12)^30^.

12 $$ GSA(C_{i} ) = \frac{{\sum\limits_{p = 1}^{P} {\left( {R(x_{i}^{p} ) - \overline{R}(x_{i}^{p} )} \right)\left( {R(t_{{}}^{p} ) - \overline{R}(t_{{}}^{p} )} \right)} }}{{\sqrt {\sum\limits_{p = 1}^{P} {\left( {R(x_{i}^{p} ) - \overline{R}(x_{i}^{p} )} \right)^{2} } } \sqrt {\sum\limits_{p = 1}^{P} {\left( {R(t_{{}}^{p} ) - \overline{R}(t_{{}}^{p} )} \right)^{2} } } }} $$

where *P* is the number of the repeated interactions; $R(x_{i}^{p} )\left( {R(t_{{}}^{p} )} \right)$ is the rank of $x_{i}^{p} \left( {t_{{}}^{p} } \right)$ among the simulated input data; $\overline{R}(x_{i}^{p} )\left( {\overline{R}(t_{{}}^{p} )} \right)$ is the mean value of $R(x_{i}^{p} )\left( {R(t_{{}}^{p} )} \right)$.

## A case study

**Figure 2:**
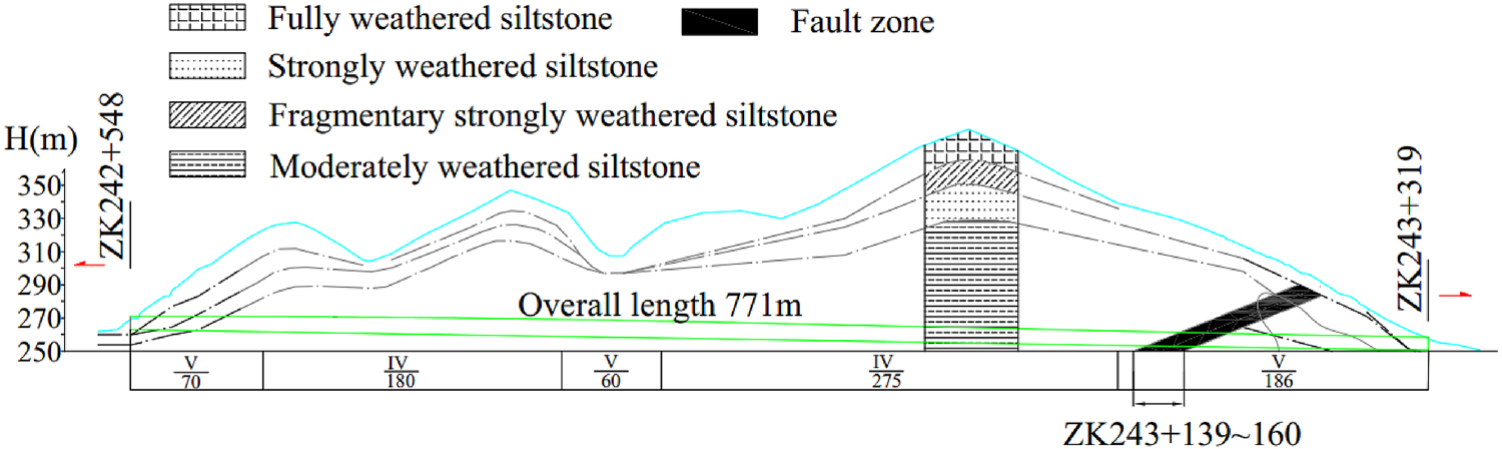
Fault structures along the Jinzhupa Tunnel (Figure no. 2 was drawn using AutoCAD software with version no. AutoCAD 2017 and link: https://www.autodesk.com.cn/).

The Jinzhupa Tunnel is a twin-tube highway tunnel. The right and left tunnels are 782 m and 771 m long, respectively. This paper takes the left line (ZK242 + 548 ~ ZK243 + 319) as the object of study. The fault structure along the left line of the tunnel is shown in Fig. 2. There are 316 m of V-level surrounding rock section and 455 m of IV-level surrounding rock section. The rock mass is mainly composed of the residual silty clay, granite fully weathered layer, and broken strong weathered layer. Furthermore, there is a fracture fragmentation zone at section ZK243 + 139 ~ 160. Affected by this, the rock mass is relatively broken, showing a huge mosaic structure or broken mosaic structure. The rock mass is broken and has varying degrees of weathering. During the construction process, it is easy to cause tunnel collapse and water burst. Therefore, it is urgent to conduct a collapse risk assessment of the tunnel to reduce the losses caused by the collapse. In the proposed fusion method, the following four steps are adopted:

Step (1) Collapse risk assessment based on statistical data: The risk mechanism of tunneling collapse is analyzed to reveal the potential risk factors. Then the collected tunneling collapse data set are used to train SVM models.

Step (2) Collapse risk assessment based on expert judgment: According to the construction personnel’s description of the site situation, the Bayesian network is used to assess the collapse risk.

Step (3) Collapse risk assessment based on monitoring data indicator: Using the arch displacement and horizontal convergence displacement monitoring data of the tunnel as the information source, the cloud model is applied for collapse risk assessment.

Step (4) Multi-source information fusion: The results of the above three assessment models are used as information sources and fused using the improved D–S theory to obtain the overall tunneling collapse risk value.

Step (5) Robustness of risk assessment results: Different percentages of deviation (5%, 10%, 15%, and 20%) were added to the collected data. The robustness of the proposed hybrid method is further validated in the presence of unavoidable data biases.

## Result and analysis

### Collapse risk assessment based statistical data

#### Risk/hazard identification in the tunnel collapse

In actual engineering, the tunneling collapse may be affected by many factors, which interact with each other. Many scholars^31–34^ have studied the risk factors of collapse and established a similar index system. Referring to previous researches, a total of 15 risk factors are selected, as shown in Table 1. The risk factors are analyzed in detail as shown in researches^2,35^. At the same time, the safety status of each tunnel collapse risk factor is divided into four levels, as shown in Table 1.

**Table 1:** Classified states of tunnel collapse risk factors.

Factors	I	II	III	IV
Tunnel collapse (*T*)	Safe	Deformation	Small-scale collapse	Large-scale collapse
Geometric factor (*B*_1_)	No risk	Low risk	Medium risk	High risk
Geological factors (*B*_2_)	No risk	Low risk	Medium risk	High risk
Construction technology (*B*_3_)	No risk	Low risk	Medium risk	High risk
Construction management factors (*B*_4_)	No risk	Low risk	Medium risk	High risk
Excavation span (m) (*X*_1_)	< 7	7–10	10–14	> 15
Depth-to-height ratio (*H*_0_/*H*) (*X*_2_)	> 20	15 ~ 20	10 ~ 15	< 10
Rock mass grade (*X*_3_)	I (81 ~ 100)	II (61 ~ 80)	III (41 ~ 60)	IV, V (< 40)
Groundwater level ((*H*_0_ + *H*)/*H*_w_) (*X*_4_)	< 5	5 ~ 20	20 ~ 35	> 35
Unfavorable geology (*X*_5_)	Non-Catastrophability (76 ~ 100)	Weak Catastrophability (51 ~ 75)	Medium Catastrophability (26 ~ 50)	Strong Catastrophability (0 ~ 25)
Bias angle (°) (*X*_6_)	< 10	10 ~ 25	25 ~ 40	> 40
Primary support stiffness (*X*_7_)	Reasonable	Almost reasonable	Unreasonable	Extremely unreasonable
Ground reinforcement measures (*X*_8_)	Accurate	Almost accurate	Inaccurate	Extremely inaccurate
Excavation method (*X*_9_)	CRD	CD	Bench	Full face
Waterproofing and drainage measures (*X*_10_)	Reasonable	Almost reasonable	Unreasonable	Extremely unreasonable
Timeliness of primary support(min) (*X*_11_)	< 30	30 ~ 60	60 ~ 120	> 120
Monitoring (*X*_12_)	Reasonable	Almost reasonable	Unreasonable	Extremely unreasonable
Construction quality (*X*_13_)	Good (76 ~ 100)	Fair (51 ~ 75)	Poor (26 ~ 50)	Very poor (0 ~ 25)
Accuracy of geological investigation (%) (*X*_14_)	> 90	75 ~ 90	60 ~ 75	< 60
Rationality of procedure linkage(*X*_15_)	Reasonable	Almost reasonable	Unreasonable	Extremely unreasonable

#### Choice of kernel function and parameters

**Figure 3:**
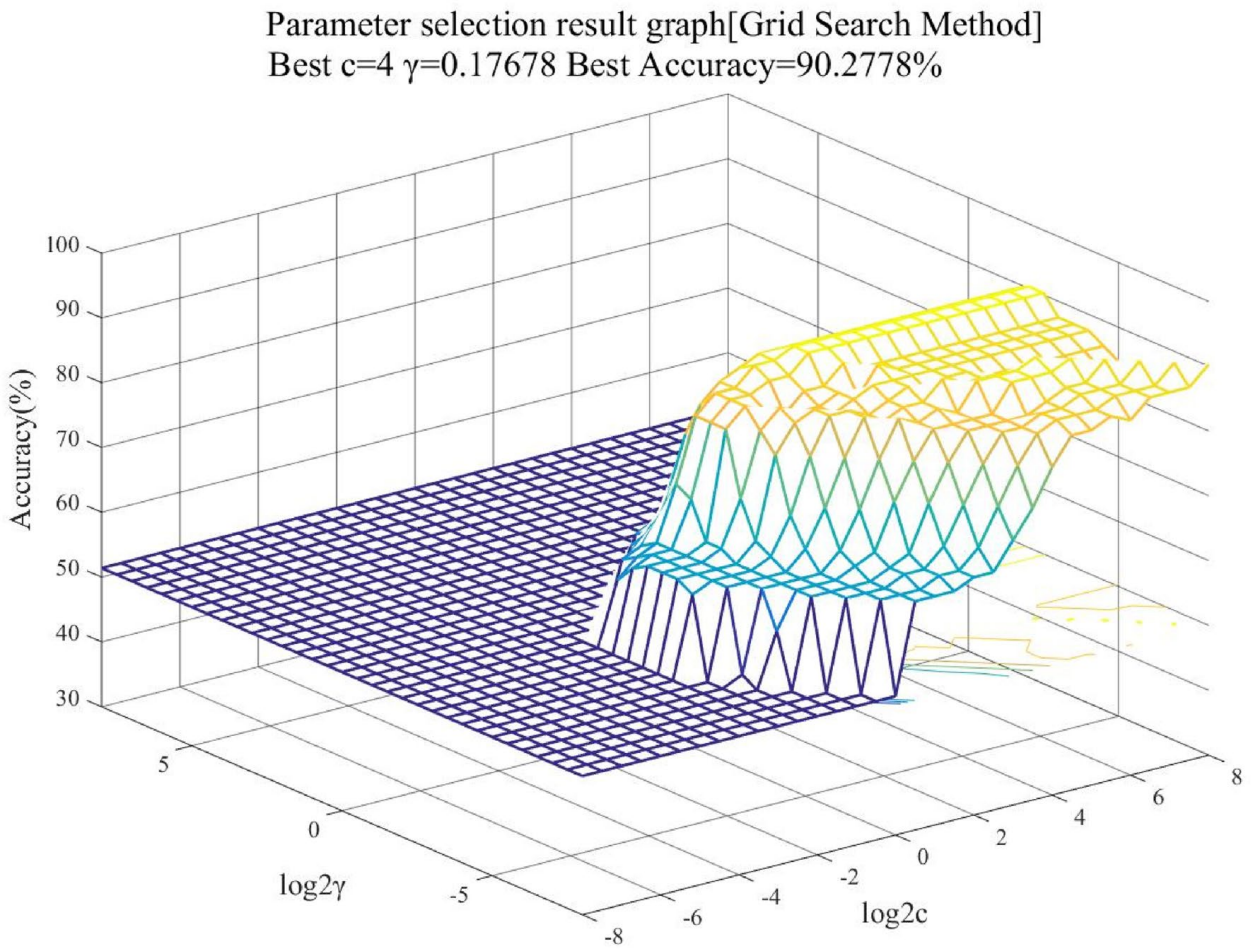
Support Vector Machines evaluation accuracy based on pairs of (*C, γ*). (Figure no. 3 was drawn using Matlab software with version no. Matlab 2020 and link: https://ww2.mathworks.cn/).

In order to construct the SVM model, a dataset of 70 tunnel collapses was collected from the study^2^ and classified according to Table 1. The dataset is used as training data, and the optimal hyperparameters (*C*, *γ*) of the SVM model are found using the grid search method. Due to the limited input data, the fivefold cross-validation is conducted to determine the best value of the penalty parameter *C* and the gamma *γ*. Pairs of (*C*, *γ*) with different values are tested in the SVM model, and their corresponding results about the classification accuracy as shown in Fig. 3. The search range for the optimal hyperparameters (*C*, *γ*) is [2^−8^, 2^8^]. When the parameter *C* = 4, *γ* = 0.17678, the accuracy of classification is the highest.

#### Calculation of the collapse risk probability

According to Eq. (6), the probability of different collapse risk levels is calculated. Since the fracture zone is prone to collapse during excavation, this paper assesses the collapse risk of the fracture zone. In tunnel sections (ZK243 + 130 ~ 330), every 10 m of the tunnel section is selected as a testing sample, and 20 samples are taken. The SVM model (*C* = 4, *γ* = 0.17678) is utilized to evaluate the collapse risk value of the testing sample(risk factor level probability distribution as input and collapse risk probability as output), the classification results as shown in Table 2. The risk level of tunnel collapse with the highest probability in the bold font in Table 2 represents the classification result. Despite the high accuracy of the probabilistic SVM evaluation results, it is worth noting that the second-highest probability is very close to the highest value in some of the prediction results. For example, a tunnel section No.9, the probability of tunnel collapse for class I (0.45) and class II (0.50) is very close, which means that the results are very uncertain.

**Table 2:** Results of probabilistic Support Vector Machines.

Tunnel section	*m* (I)	*m* (II)	*m* (III)	*m* (IV)	Predicted risk	Ture risk
No.1	0.14	**0.60**	0.18	0.08	II	II
No.2	0.02	0.02	**0.94**	0.02	III	III
No.3	0.04	**0.91**	0.03	0.01	II	II
No.4	**0.64**	0.30	0.06	0.00	I	II
No.5	0.04	**0.91**	0.03	0.02	II	II
No.6	0.03	**0.91**	0.04	0.02	II	II
No.7	0.07	**0.87**	0.04	0.02	II	II
No.8	**0.91**	0.03	0.03	0.03	I	I
No.9	0.45	**0.50**	0.02	0.03	II	I
No.10	**0.83**	0.09	0.04	0.04	I	I
No.11	0.03	**0.89**	0.05	0.03	II	II
No.12	0.01	**0.93**	0.01	0.05	II	III
No.13	**0.87**	0.08	0.03	0.02	I	I
No.14	**0.92**	0.04	0.02	0.02	I	I
No.15	0.05	**0.91**	0.02	0.01	II	II
No.16	**0.87**	0.08	0.02	0.02	I	I
No.17	0.05	**0.90**	0.03	0.02	II	II
No.18	**0.86**	0.08	0.03	0.03	I	I
No.19	**0.91**	0.03	0.03	0.03	I	I
No.20	0.03	**0.91**	0.04	0.02	II	II

Significant values are in bold.

### Collapse risk assessment based on expert judgment

#### Establishment of the DAG and CPT

**Figure 4:**
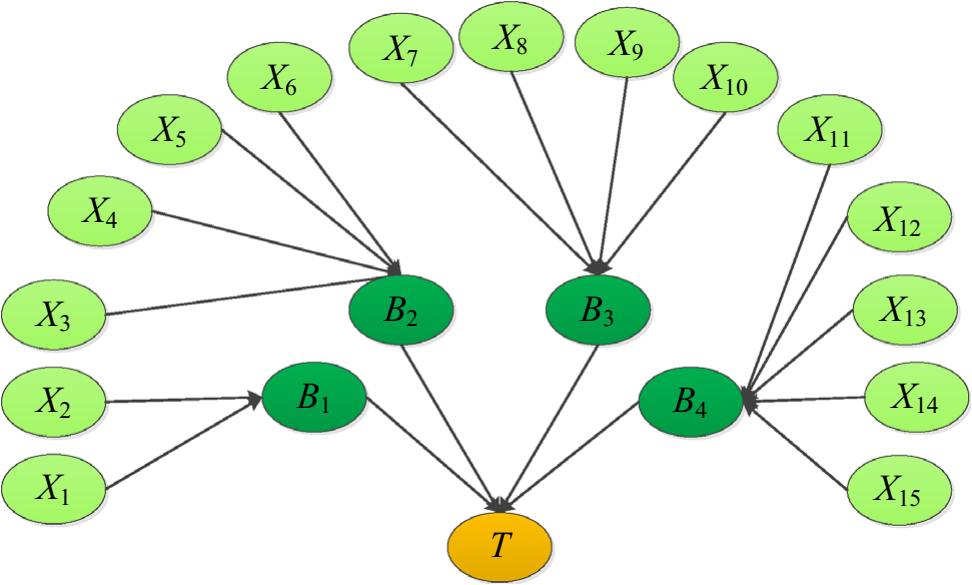
DAG of Bayesian network.

The DAG is mainly constructed by directed edges and node variables that represent the probability causality between node variables. In combination with the risk factors in Table 1, the DAG can be established, as shown in Fig. 4. To reduce the uncertainty of expert judgment, an expert survey based on confidence index is used to construct the conditional probability tables (CPT), the detail as seen in the research^34^.

#### Calculation of the tunneling collapse risk

Similarly, taking the above 20 sections as examples, experts were invited to rate the risk factors in Table 1. The each risk score value is entered into CPT, and then the probability distribution of collapse risk values is obtained by Eq. (8), the result as shown in Table 3.

**Table 3:** Results of Bayesian network at ten monitoring sections.

Tunnel section	*m* (I)	*m* (II)	*m* (III)	*m* (IV)	Predicted level	Ture level
No.1	0.00	0.48	**0.52**	0.00	III	II
No.2	0.00	0.00	**0.93**	0.07	III	III
No.3	0.00	**0.70**	0.30	0.00	II	II
No.4	0.00	**0.96**	0.04	0.00	II	II
No.5	0.00	**0.96**	0.04	0.00	II	II
No.6	**0.78**	0.22	0.00	0.00	I	II
No.7	0.00	**0.97**	0.03	0.00	II	II
No.8	**0.84**	0.16	0.00	0.00	I	I
No.9	0.19	**0.81**	0.00	0.00	II	I
No.10	**0.87**	0.13	0.00	0.00	I	I
No.11	0.00	**0.99**	0.01	0.00	II	II
No.12	0.00	0.01	**0.83**	0.17	III	III
No.13	**0.96**	0.03	0.01	0.00	I	I
No.14	**0.65**	0.35	0.00	0.00	I	I
No.15	0.07	**0.93**	0.00	0.00	II	II
No.16	0.33	**0.67**	0.00	0.00	II	I
No.17	0.01	**0.98**	0.00	0.00	II	II
No.18	**0.66**	0.33	0.01	0.00	I	I
No.19	0.18	**0.82**	0.00	0.00	II	I
No.20	0.00	**0.93**	0.07	0.00	II	II

Significant values are in bold.

Compared with the prediction results of the probability SVM, the accuracy of the model is lower. The second-highest probability in some prediction results is also very close to the highest value. For example, a tunnel section No.1, the probability of tunnel collapse for class II (0.48) and class III (0.52) is very close, which means that the results are very uncertain.

### Collapse risk assessment based on monitoring data indicator

#### Monitoring data indicator system

The monitoring measurement data includes the displacement of the vault, the surface settlement of the shallow buried section, and the change of the surrounding rock convergence. These data can reflect the stability of the tunnel support after the initial lining, thereby assessing the risk of collapse. Combined with this project, the vault displacement and the convergence displacement are used to analyze the collapse risk. According to the Chinese standards “Technical code for monitoring measurement of highway tunnel (DB 35/T 1067-2010)” and “Technical specification for construction of highway tunnel (JTG/T 3660-2020)”, the daily deformation rate and cumulative deformation of the two-monitoring data are divided into four levels, as shown in Table 4 where, the cumulative deformation (*y*) should be multiplied by the coefficient (*ζ*) according to the distance between the measuring point and the excavation surface (*D*), the detail as shown in Table 5where *B* is the face span of the excavation section.

**Table 4:** Classified states of monitoring measurement data.

Tunnel collapse level	I (safe)	II (deformation)	III (small-scale collapse)	IV (large-scale collapse)
Daily deformation rate (mm/day)	0 ≤ *x* < 2	2 ≤ *x* < 5	5 ≤ *x* < 10	10 ≤ *x* ≤ 20
Cumulative deformation (mm)	0 ≤ *y* < 50	50 ≤ *y* < 100	100 ≤ *y* < 200	200 ≤ *y* ≤ 300

**Table 5:** The coefficient (*ζ*) of the cumulative deformation (*y*).

The distance between the measuring point and the excavation surface (*D*)	1*B*	2*B*	*3B*	4*B* ~ 4*B*
*ζ*	0.5	0.75	0.85	1

#### Monitoring data collection

**Figure 5:**
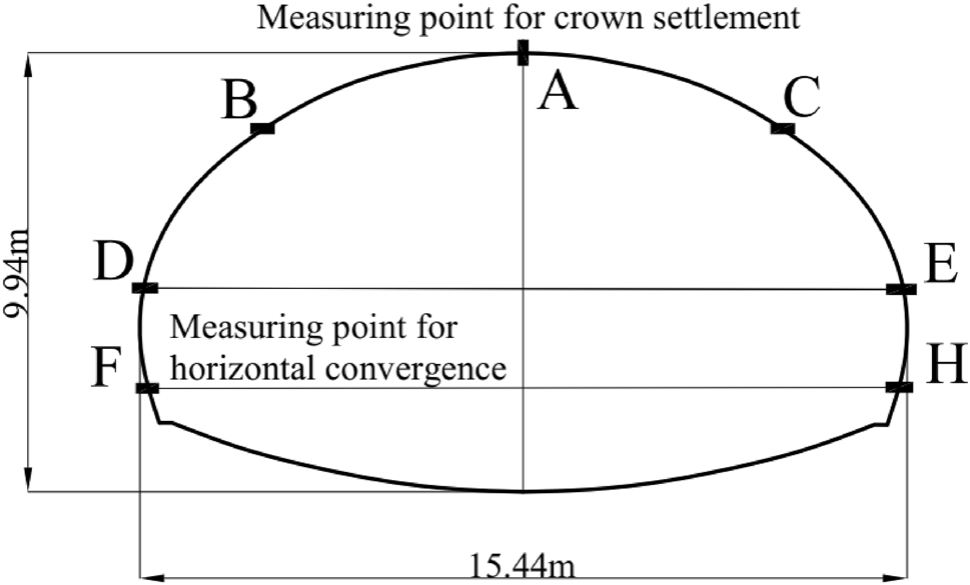
Schematic diagram of monitoring point layout.

The tunnel is excavated by the bench method, and the monitoring points and measuring points are arranged as shown in Fig. 5. Among them, point A, B, and C are the monitoring points for the settlement of the vault, DE and FH are the surrounding rock convergence line. The surrounding rock displacement is monitored once in the morning and once in the evening, and the average value is taken as the monitoring value of the day.

#### Calculation of the tunneling collapse risk

According to Eq. (3), the cloud model parameter values $\left( {Ex,En,He} \right)$ of the two monitoring indicators are constructed, as shown in Table 6. Finally, the tunnel collapse risk BPAs is constructed by Eq. (4).

**Table 6:** Cloud models parameter value of the two monitoring indicators.

Indicators	I			II			III			IV		
	*Ex*	*En*	*He*	*Ex*	*En*	*He*	*Ex*	*En*	*He*	*Ex*	*En*	*He*
Daily settlement	1	0.333	0.002	3.5	0.5	0.002	7.5	0.833	0.002	12.5	0.833	0.002
Cumulative settlement	25	8.333	0.002	75	8.333	0.002	150	16.777	0.002	250	16.777	0.002

**Figure 6:**
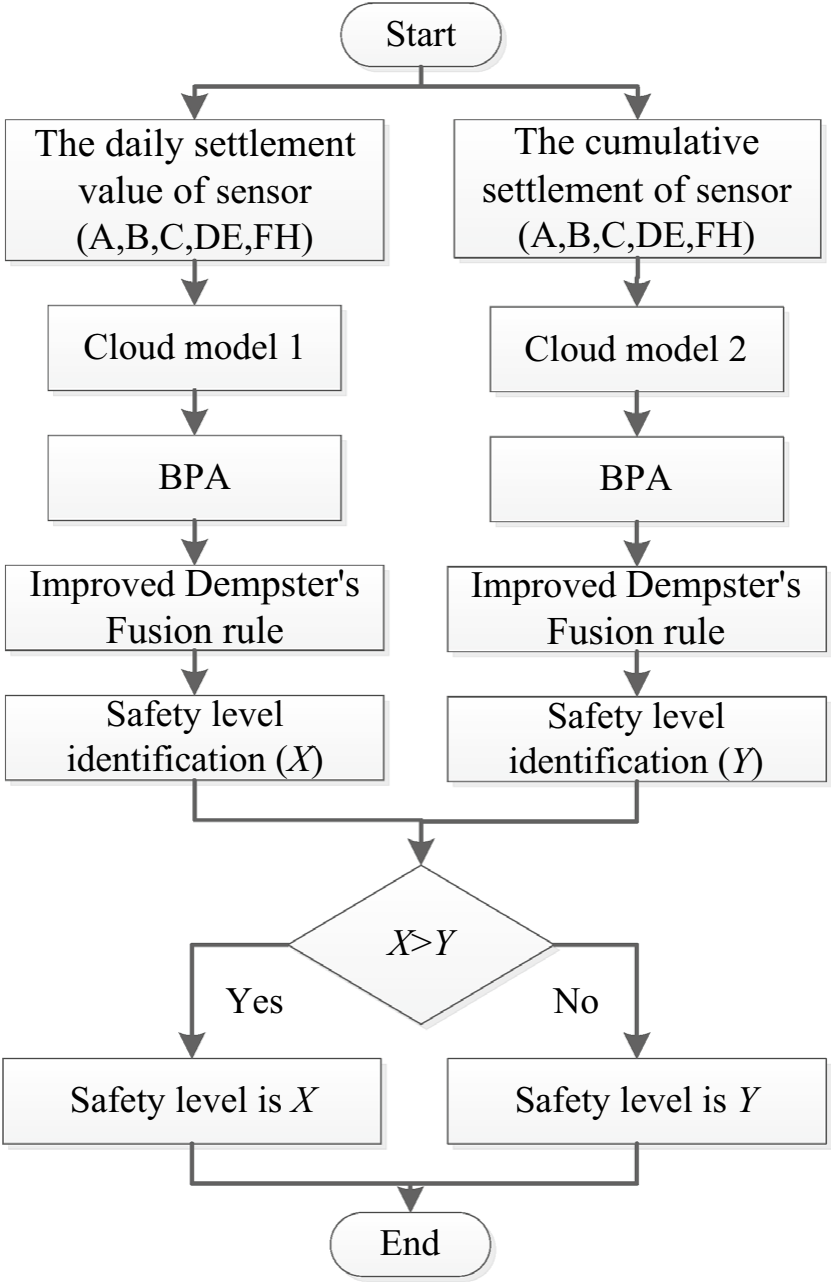
Flowchart of monitoring data processing.

The cloud model is used to obtain the BPAs of the cumulative settlement and daily settlement of the monitoring data (A, B, C, DE, and FH), and the improved D–S theory is used to fuse them separately to obtain the risk level. Finally, the maximum value of the two result is selected as the collapse risk value, the flowchart as shown in Fig. 6.

According to the flowchart shown in Fig. 6, The monitoring data from the above 20 sections were used for collapse risk assessment. According to Eq. (4), the monitoring data are converted into risk probability distribution values, the result as shown in Table 7. Obviously, in tunnel sections (No.6 and No.9), the probability of tunnel collapse for class I (0.50) and class II (0.50) is very close, which means that the results are very uncertain. It is difficult to make an accurate judgment on the risk of tunnel collapse.

**Table 7:** Results of Cloud model at ten monitoring sections.

Tunnel section	*m* (I)	*m* (II)	*m* (III)	*m* (IV)	*m* (Θ)	Predicted level	Ture level
No.1	**0.52**	0.46	0.00	0.00	0.02	I	II
No.2	0.00	0.00	**1.00**	0.00	0.00	III	III
No.3	0.48	**0.50**	0.00	0.00	0.02	II	II
No.4	0.00	**0.95**	0.00	0.00	0.05	II	II
No.5	0.01	**0.97**	0.00	0.00	0.02	II	II
No.6	**0.50**	**0.50**	0.00	0.00	0.00	–	II
No.7	0.00	**1.00**	0.00	0.00	0.00	II	II
No.8	**1.00**	0.00	0.00	0.00	0.00	I	I
No.9	**0.50**	**0.50**	0.00	0.00	0.00	–	I
No.10	0.00	**0.99**	0.00	0.00	0.01	II	I
No.11	0.41	**0.53**	0.00	0.00	0.06	II	II
No.12	0.00	**0.60**	0.35	0.00	0.05	II	III
No.13	**0.96**	0.00	0.00	0.00	0.04	I	I
No.14	**1.00**	0.00	0.00	0.00	0.00	I	I
No.15	0.03	**0.90**	0.00	0.00	0.07	II	II
No.16	0.58	**0.40**	0.00	0.00	0.02	II	I
No.17	0.00	**0.93**	0.00	0.04	0.03	II	II
No.18	**1.00**	0.00	0.00	0.00	0.00	I	I
No.19	**0.98**	0.00	0.00	0.00	0.02	I	I
No.20	0.00	**1.00**	0.00	0.00	0.00	II	II

Significant values are in bold.

### Multi-source information fusion

In order to settle the problem of unreliable evaluation results of single-information sources, the improve D–S evidence theory (section 2.4) is used to fuse the multi-source data. This method combines the different results of the three above-mentioned single-source assessment methods. According to Eqs. (9) and (10), the fusion results can be calculated, the result as shown in Table 8. To demonstrate the effectiveness of the new fusion method, several sections with conflicting information were selected for comparison with the traditional D–S theory, as shown in Table 9. The following conclusions can be obtained:

1. The multiple-information fusion method proposed in this paper can improve the accuracy and reduce uncertainty in the tunnel collapse risk evaluation. Only section evaluation error appears at section No. 9, indicating that the evaluation accuracy rate of 20 sections has reached 95%. The confidence indexes *m*(Θ) of the 20 tunnel sections are all close to 0, which means that the uncertainty of the results is 0.
2. The proposed method can solve the problem of inconsistent results of the three risk assessment methods effectively. For example, because the results of the three risk categories are different (SVM and CM belong to level I and BN belong to level II), the single-source risk assessment method cannot directly assess the overall tunnel collapse risk level of the monitoring section 4. The multi-source information fusion method is used to evaluate the monitoring section 4 and the results are shown in Table 8. The BPAs value of tunnel collapse risk level II (that is *m* (II)) is equal to 1, which means that the collapse risk level for the monitoring section 4 is level II with a high confidence level
3. When the evaluation results of three single information sources are different (e.g. Tunnel section No.1 and No.12), the fusion result of the improve D–S theory is better than the tradional D–S theory. Dempster’s rule accumulates consensus support only and rejects a proposition completely if it is opposed by any evidence, no matter what support it may get from any other evidence. As a result, when three kinds of single information evaluation give different results, the tradional D–S theory will give a fusion result contrary to common sense. The improve D–S theory has high accuracy when merging high conflict information sources because it combines the weighted mean rule.

**Table 8:** Results of multi-source information fusion at ten monitoring sections.

Tunnel section	*m* (I)	*m* (II)	*m* (III)	*m* (IV)	*m* (Θ)	Predicted level	Ture level
No.1	0.00	**0.99**	0.01	0.00	0.00	II	II
No.2	0.00	0.00	**1.00**	0.00	0.00	III	III
No.3	0.00	**1.00**	0.00	0.00	0.00	II	II
No.4	0.00	**1.00**	0.00	0.00	0.00	II	II
No.5	0.00	**1.00**	0.00	0.00	0.00	II	II
No.6	0.00	**1.00**	0.00	0.00	0.00	II	II
No.7	0.00	**1.00**	0.00	0.00	0.00	II	II
No.8	**1.00**	0.00	0.00	0.00	0.00	I	I
No.9	0.17	**0.83**	0.00	0.00	0.00	II	I
No.10	**0.85**	0.11	0.02	0.02	0.00	I	I
No.11	0.00	**1.00**	0.00	0.00	0.00	II	II
No.12	0.00	0.35	**0.64**	0.01	0.00	III	III
No.13	**1.00**	0.00	0.00	0.00	0.00	I	I
No.14	**1.00**	0.00	0.00	0.00	0.00	I	I
No.15	0.00	**1.00**	0.00	0.00	0.00	II	II
No.16	**0.88**	0.12	0.00	0.00	0.00	I	I
No.17	0.00	**1.00**	0.00	0.00	0.00	II	II
No.18	**1.00**	0.00	0.00	0.00	0.00	I	I
No.19	**1.00**	0.00	0.00	0.00	0.00	I	I
No.20	0.00	**1.00**	0.00	0.00	0.00	II	II

Significant values are in bold.

**Table 9:** Comparison of fusion methods.

Tunnel section	Evaluation model	Probability over Class I	Probability over Class II	Probability over Class III	Probability over Class IV	Predicted label	True label
No.1	E1	0.14	**0.6**	0.18	0.08	II	II
	E2	0.00	0.48	**0.52**	0.00	III	II
	E3	**0.52**	0.46	0.00	0.00	I	II
Fusion	Improve	0.00	**0.99**	0.01	0.00	II	II
	Traditonal	**0.65**	0.35	0.00	0.00	I	II
No.2	E1	0.02	0.02	**0.94**	0.02	III	III
	E2	0.00	0.00	**0.93**	0.07	III	III
	E3	0.00	0.00	**1.00**	0.00	III	III
Fusion	Improve	0.00	0.00	**1.00**	0.00	III	III
	Traditonal	0.00	0.00	**1.00**	0.00	III	III
No.3	E1	0.04	**0.91**	0.03	0.01	II	II
	E2	0.00	**0.70**	0.30	0.00	II	II
	E3	0.48	**0.50**	0.00	0.00	II	II
Fusion	Improve	0.00	**1.00**	0.00	0.00	II	II
	Traditonal	0.00	**1.00**	0.00	0.00	II	II
No.4	E1	**0.64**	0.30	0.06	0.00	I	II
	E2	0.00	**0.96**	0.04	0.00	II	II
	E3	0.00	**0.95**	0.05	0.00	II	II
Fusion	Improve	0.00	**1.00**	0.00	0.00	II	II
	Traditonal	0.00	**1.00**	0.00	0.00	II	II
No.12	E1	0.01	**0.93**	0.01	0.05	II	III
	E2	0.00	0.01	**0.83**	0.17	III	III
	E3	0.00	**0.64**	0.36	0.00	II	III
Fusion	Improve	0.00	0.35	**0.64**	0.01	III	III
	Traditonal	0.10	**0.90**	0.10	0.00	II	III

Significant values are in bold.

### Verification of evaluation results

**Figure 7:**
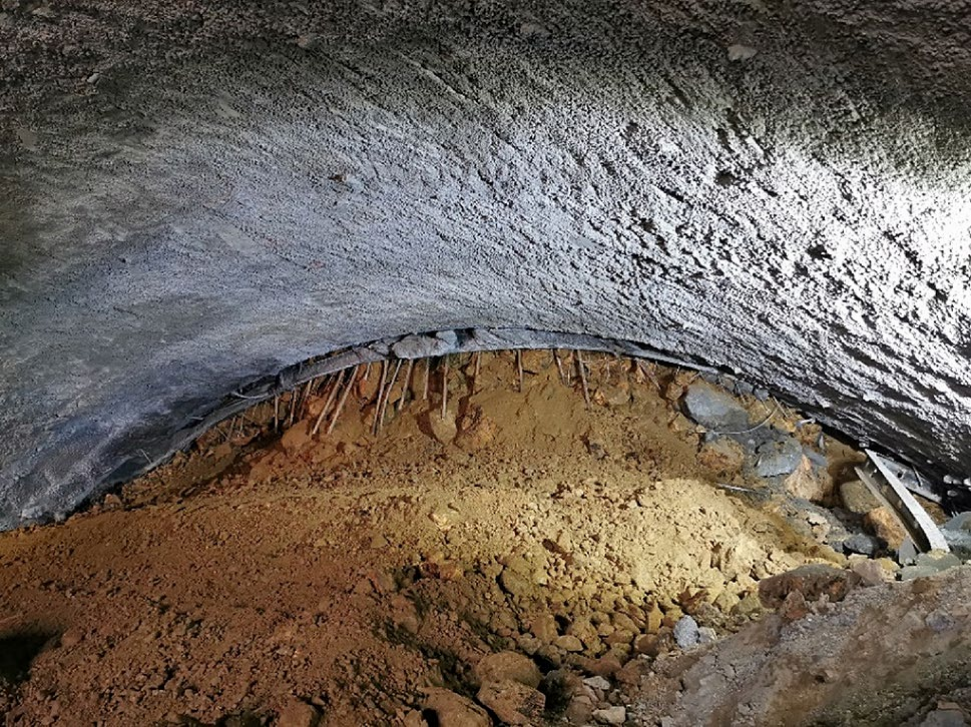
Tunnel collapse.

When the tunnel was excavated to section ZK243 + 143, the tunnel vault collapsed, as shown in Fig. 7. This is due to the section being in the fracture zone of the surrounding rock and the insufficient strength of the tunnel lining support, resulting in the tunnel collapse. The multi-source information fusion assessment method was applied to this section for collapse risk assessment, the results as shown in Table 8 (No.2 tunnel section). The results indicate that the section is at small-scale collapse risk with a probability of 1. This section is likely to occur a small-scale collapse if the support conditions are not strengthened. The tunneling collapse risk assessment results are consistent with reality, which proves the usefulness of the assessment method in the actual construction process.

### Robustness of risk assessment results

**Figure 8:**
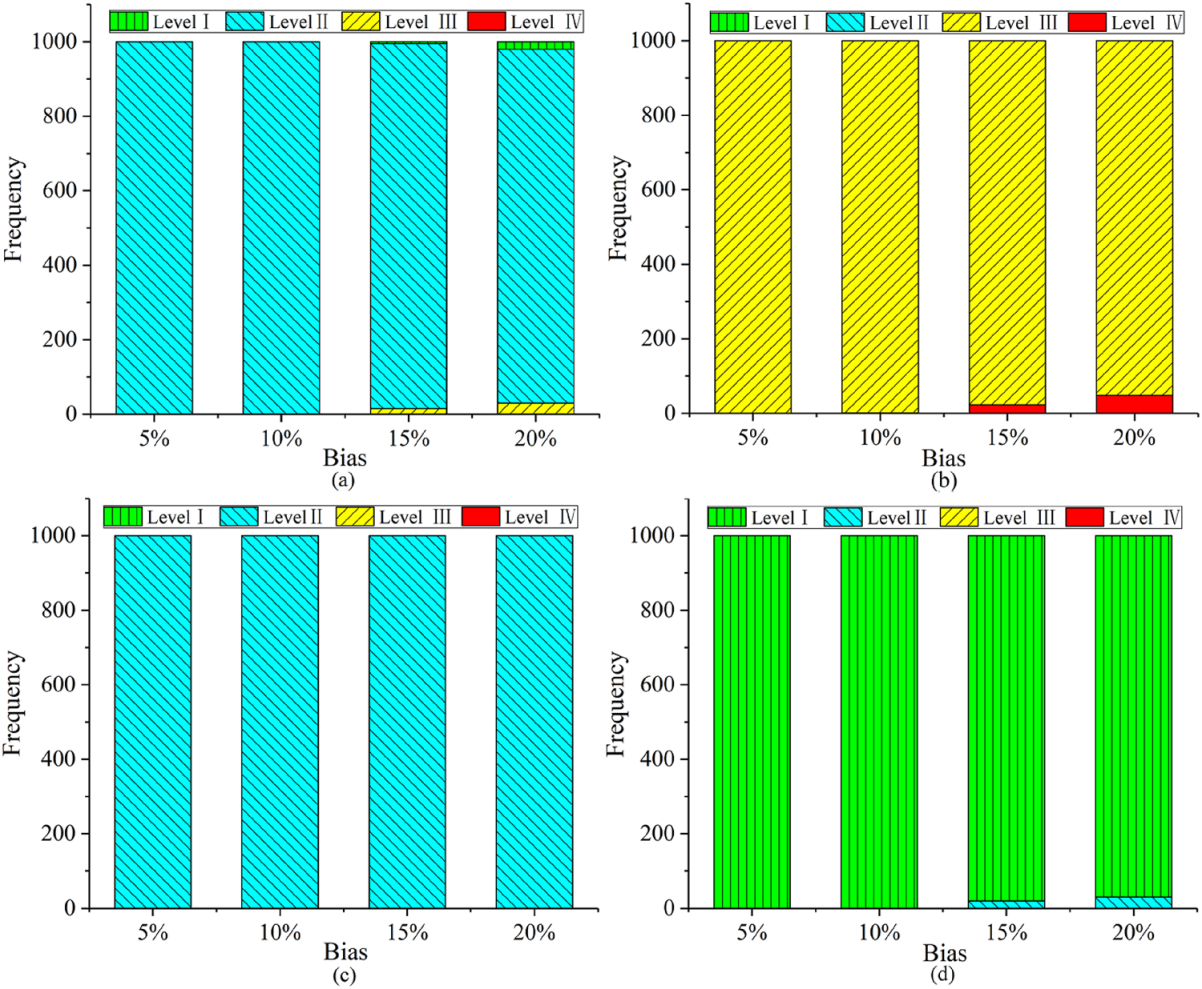
Tunnel collapse risk assessment results after 1000 iterations under different deviation levels at four section: (**a**) No.1; (**b**) No.2; (**c**) No.3; (**d**) No.8.

**Figure 9:**
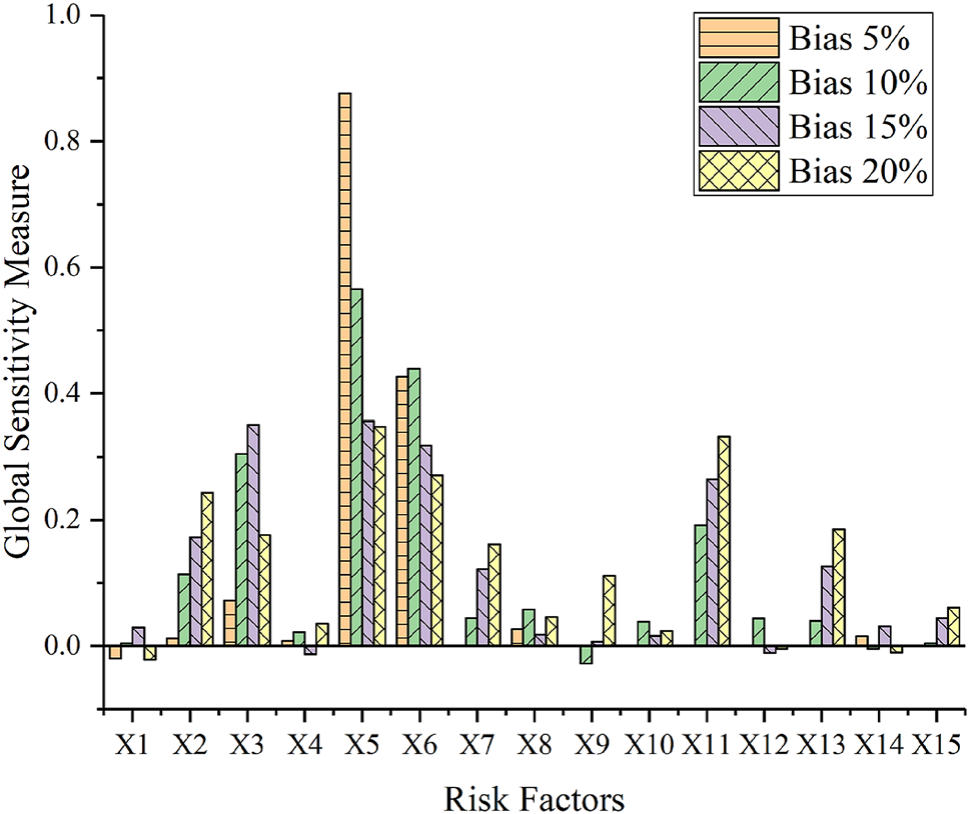
Global sensitivity analysis of 15 risk indicators (No. 2 section).

In actual engineering, due to the influence of measurement errors and human factors, data from multi-source observations may have inevitable deviations. This article will use the MC simulation technology to simulate the uncertainty of the data. The factors affecting tunnel collapse are assumed to obey normal distribution. To further verify the robustness of the proposed hybrid method under unavoidable deviations, we added different deviation percentages (i.e. 5%, 10%, 15%, and 20%) to the collected data. In this paper, the number of repeated iterations *P* is set to 1000. Figure 8 shows the results of tunnel collapse risk assessment after 1000 iterations for 4 tunnel sections (No. 1, 2, 3, and 8) at different offset levels. Figure 9 shows the global sensitivity analysis about tunnel section No. 2. The following conclusions can be obtained:

1. The proposed multi-source information fusion approach has good robustness to deviation. In order to better understand the bias, Fig. 8 shows the frequency of a certain collapse risk level after 1000 iterations under different biases in 4 tunnel sections (No.1, No.2, No.3, and No.8). When the percentage of bias is increased, the accuracy of the risk assessment will be slightly reduced, but it will remain at a high level. Obviously, all data with a deviation of less than 10% can almost achieve an evaluation accuracy rate close to 100%, proving that the method is accurate and reliable under low bias. When the deviation is 20%, the evaluation accuracy of all tunnel sections is still higher than 90%, which proves that the method has strong robustness under high deviations. Anymore, the accuracy of the assessment of the No. 3 tunnel section under each level of bias has reached 100%. This is because the results of the three single-information evaluation methods of tunnel section No. 3 are consistent [that is, the results of all three different models are risk level II (Deformation)], so no conflicting information will have a negative impact on the result of multi-source information fusion.
2. Since the No. 2 tunnel section is in a dangerous state (Small-scale collapse), a global sensitivity analysis is performed on this part to find out the key risk factors that affect the tunnel collapse. Therefore, some measures to prevent tunnel collapse can be taken in advance. The Spearman’s rank correlation coefficient [Eq. (12)] is used to measures the degree of influence of risk factors on the risk level of the tunneling collapse. As shown in Fig. 9, *X*_3_, *X*_5_, *X*_6_, and *X*_11_ are the top four risk factors that have the greatest impact on tunnel collapse. To reduce the risk level of tunnel section No.2, more attention should be paid to these four risk factors. In addition, when the deviation level increases to 20%, the results of the most sensitive risk factors remain unchanged, again verifying the robustness of the proposed method.

## Discussion

**Figure 10:**
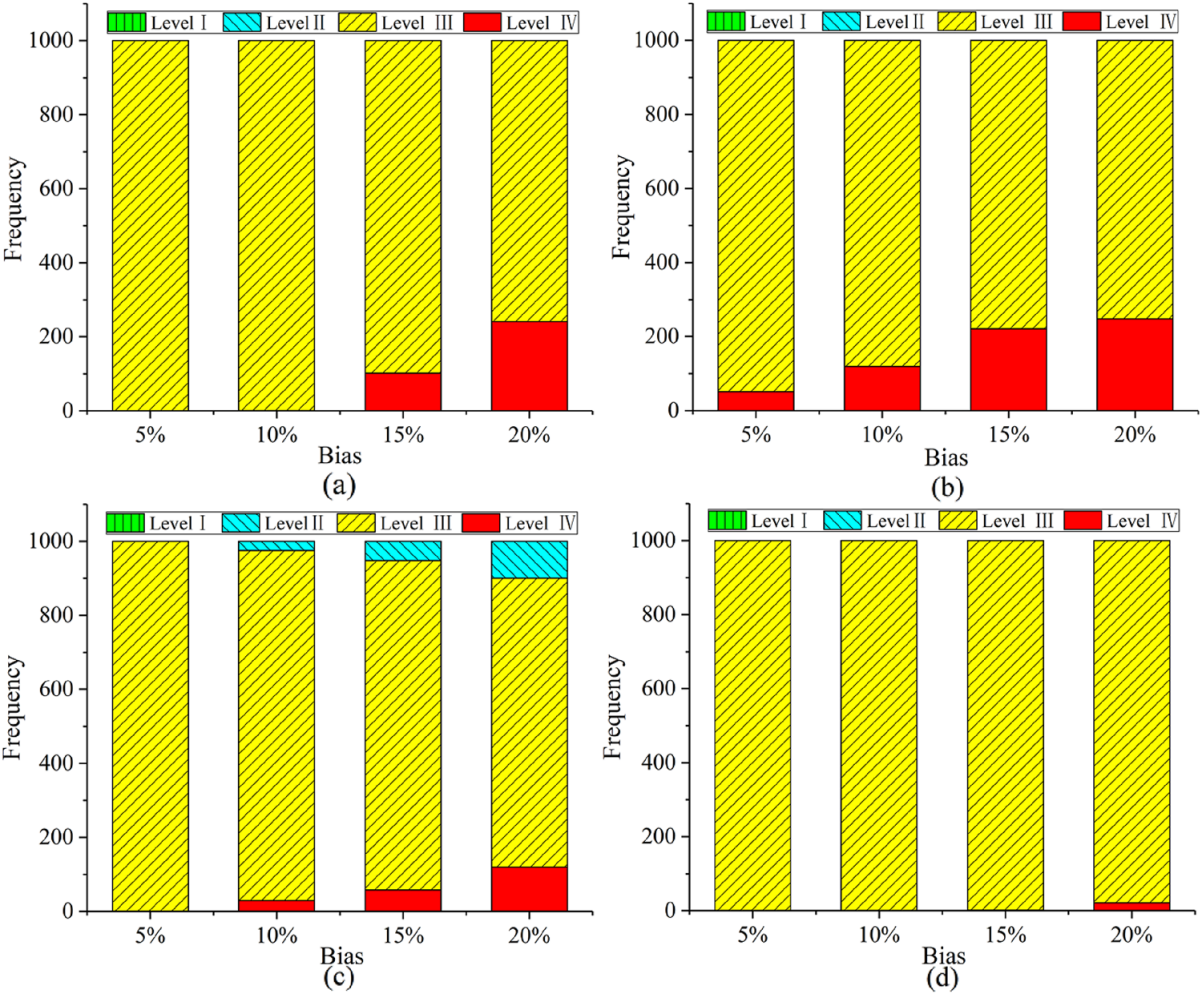
Four risk evaluation methods for tunnel collapse risk assessment after 1000 iterations under different deviation levels: (**a**) SVM; (**b**) BN; (**c**) CM; (**d**) Multi-source information fusion method.

There is no doubt that the single-source information assessment method also can estimate the tunnel collapse risk level. However, the single source of information does not fully reflect the environment of the tunnel construction, resulting in a certain bias and low accuracy of the assessment results. To compare the single-source information evaluation method with the multi-source information fusion method, the Monte Carlo simulation is used to simulate the inevitable uncertainty, and the four evaluation methods are calculated 1000 times. The tunneling collapse risk assessment results of four assessment methods iterate 1000 times under different deviation levels at tunnel section No.2, as shown in Fig. 10. The following conclusions can be obtained:

The single-source information assessment method (Fig. 10a–c) can get an accurate assessment result in case of small deviations, but it performs poorly at high bias. The multi-source information fusion method is more robust than the single-source information assessment method. As seen in Fig. 10d, the multi-source information fusion method has a higher accuracy of assessment under a large bias, proving that the proposed method has good robustness. This is because the proposed method makes full use of available information, including contradictory information. When the data deviation is 20%, the evaluation accuracy of the single-source information evaluation method is less than 80% in 1000 iterations. In order words, the single-source information assessment method has a high sensibility to bias. However, the multi-source information fusion method can still have 97.9% accuracy of assessment in a 20% bias. This method is a good solution to the data bias caused by the large amount of uncertainty and complexity of the underground environment.

## Conclusions and future works

This paper proposes a multi-source information fusion method for the tunneling collapse risk assessment, which provides risk warning and decision-making suggestions for tunnel excavation. The analysis process consists of four main steps: (1) Risk assessment systems are established for the three information sources (i.e. statistical data, physical sensors, and expert judgment provided by humans) separately; (2) The three information sources are processed by the BN, CM, and SVM respectively to obtain the BPAs of the collapse risk; (3) All predictions from three different assessment method are fused to obtain the overall tunneling collapse risk; (4) The Monte Carlo simulation method is used for global sensitivity analysis and robustness verification. Finally, the Jinzhupa tunnel in China is used to verify the applicability of the proposed approach. The methods developed in this research have the following innovations and capabilities:

1. It can synthesize multi-source information to obtain a more accurate result for the tunneling collapse risk assessment. Due to many risk factors, the tunneling collapse risk assessment is a multi-attribute decision-making problem. In this paper, both soft data from domain experts and hard data from electrical sensors and statistical data are used for evaluating the tunnel collapse risk. A hybrid combination rule combining the weighted mean rule and Dempster’s rule is proposed to process multiple conflicting pieces of evidence. Besides, a confidence index, *m*(Θ) is adopted to measure the reliability of the tunnel collapse risk result. As shown in Table 8, the value of *m*(Θ) is zero, indicating that the tunneling collapse risk has a high degree of confidence.
2. As the deviation level of input data increases, the accuracy rate of the single-source information evaluation method is gradually decreasing. However, the proposed multi-source information fusion method is very robust to deviations. Even when the deviation is 20%, the accuracy of the collapse risk assessment still reaches 97.9%. In other words, this method has excellent tolerance to bias, which eliminates the adverse effects of deviation to the maximum extent and ensures the accuracy and reliability of the evaluation results.
3. When the tunnel section is in a dangerous state, in order to provide advice to decision-makers, global sensitivity analysis is proposed to identify the most influential risk factors. The global sensitivity analysis considers the interaction between risk factors, making the results more in line with actual construction conditions. In the tunnel case of this study, the factors *X*_3_ (Rock mass grade), *X*_5_ (Unfavorable geology), *X*_6_ (Bias angle), and *X*_11_ (Timeliness of primary support) are identified to have the greatest impact on the risk of tunnel collapse. Besides, because measurement errors or human errors may cause data deviations, the MC method is used to simulate the data deviations to prove that the proposed method still has good robustness under deviations.

The proposed method in this paper also has some limitations. Experts are still required to participate in the entire evaluation process, which means that a truly automated evaluation has not yet been achieved. In terms of tunnel collapse data collection, the amount of data is still small, and a system needs to be developed to collect data on a global scale. In addition, this method cannot predict the risk status of the next construction process, and further research is needed.

## Data Availability

The datasets generated during and/or analyzed during the current study are not publicly available but are available from the corresponding author on reasonable request.
